# Clay Components in Soil Dictate Environmental Stability and Bioavailability of Cervid Prions in Mice

**DOI:** 10.3389/fmicb.2016.01885

**Published:** 2016-11-23

**Authors:** A. Christy Wyckoff, Sarah Kane, Krista Lockwood, Jeff Seligman, Brady Michel, Dana Hill, Aimee Ortega, Mihnea R. Mangalea, Glenn C. Telling, Michael W. Miller, Kurt Vercauteren, Mark D. Zabel

**Affiliations:** ^1^Department of Microbiology, Immunology and Pathology, College of Veterinary Medicine and Biomedical Sciences, Prion Research Center at Colorado State UniversityFort Collins, CO, USA; ^2^Colorado Parks and WildlifeFort Collins, CO, USA; ^3^National Wildlife Research Center, Wildlife Services, United States Department of AgricultureFort Collins, CO, USA

**Keywords:** chronic wasting disease (CWD), prions, soil, clay, environment, transmission, montmorillonite, prion trafficking

## Abstract

Chronic wasting disease (CWD) affects cervids and is the only known prion disease to affect free-ranging wildlife populations. CWD spread continues unabated, and exact mechanisms of its seemingly facile spread among deer and elk across landscapes in North America remain elusive. Here we confirm that naturally contaminated soil contains infectious CWD prions that can be transmitted to susceptible model organisms. We show that smectite clay content of soil potentiates prion binding capacity of different soil types from CWD endemic and non-endemic areas, likely contributing to environmental stability of bound prions. The smectite clay montmorillonite (Mte) increased prion retention and bioavailability *in vivo*. Trafficking experiments in live animals fed bound and unbound prions showed that mice retained significantly more Mte-bound than unbound prions. Mte promoted rapid uptake of prions from the stomach to the intestines via enterocytes and M cells, and then to macrophages and eventually CD21+ B cells in Peyer's patches and spleens. These results confirm clay components in soil as an important vector in CWD transmission at both environmental and organismal levels.

## Introduction

Chronic Wasting Disease is a transmissible spongiform encephalopathy (TSE), or prion disease, that affects cervid species including elk (*Cervis elaphus nelsoni*), mule deer (*Odocoileus hemionus*), white-tailed deer (*O. virginianus*), moose (*Alces alces spp*.) and reindeer (*Rangifer tarandus tarandus*). CWD has been horizontally transmitted directly between animals (Miller and Williams, 2003), through excreta (Mathiason et al., 2006), and through indirect transmission from contaminated environments and fomites (Georgsson et al., 2006; Mathiason et al., 2009). This is similar to scrapie, a prion disease of sheep. Both diseases appear to be lymphotropic, orally transmissible, and environmentally stable (Georgsson et al., 2006; Michel et al., 2012b).

Exact routes of host exposure to CWD prions in the environment have not been entirely elucidated, but their presence in the excreta and tissues of infected animals suggests continued prion deposition into the environment while animals live, and final deposition of total prion loads when animals eventually die and decompose (Angers et al., 2009; Tamgüney et al., 2009; Haley et al., 2011; Pulford et al., 2012). Incidental oral exposure to these environmental deposits may lead to transmission in susceptible species. Indirect transmission becomes relevant when considering the browsing and grazing habits, mineral lick use, territorial marking and social grooming behaviors of cervid species (Vercauteren et al., 2007a,b; Walter et al., 2011). A likely route of transmission is through intentional and unintentional geophagy, which is common among many animals (Arthur and Alldredge, 1979; Beyer et al., 1994; Hui, 2013). Mule deer consume between 7 and 30 g of soil/day (Arthur and Alldredge, 1979), creating a probable link in the indirect CWD transmission cycle.

Perhaps equally important, the phenomenal stability of CWD and scrapie prions has led to documented persistence, in and on, fomites and the environment (Miller et al., 2004; Georgsson et al., 2006; Seidel et al., 2007; Maddison et al., 2010). Johnson et al., using hamster adapted scrapie prions, demonstrated the tight adsorption that occurs between the infectious scrapie prion molecule, PrP^Sc^, and different soil particles (Johnson et al., 2006) and that soil-bound PrP^Sc^ had increased infectivity when orally inoculated (Johnson et al., 2007). However, the pathobiological mechanisms that increase infectivity of soil-bound prions, and whether soil or its components similarly bind CWD prion molecules (PrP^CWD^) remain unexplored.

The quantity and persistence of PrP^CWD^ in the environment has been a particularly challenging aspect to study due to limited sensitivity of existing laboratory assays. Experimental evidence support indirect CWD transmission from environmental contamination to previously unexposed mule deer in a period of less than 1 year (Miller et al., 2004). While deer used in that study were captured from areas where no CWD had been detected, relatively insensitive immunohistochemistry, currently the gold standard diagnostic test for TSEs was used for CWD determination. Highly sensitive prion detection assays had not yet been developed to detect low-level subclinical infection or to estimate environmental prion loads. Furthermore, soil as the source of prion contamination was not directly assessed, and other fomites such as birds or rodents could not be definitively excluded.

Experimental studies of indirect transmission of CWD from environmental PrP^CWD^ contamination are required to understand the disease ecology, epidemiology, pathology and overall maintenance of CWD in wild populations. Assessing the impact of environmental prions in new CWD infections requires following the fate of soil-bound prions once ingested by animals. We now have the tools to address these and other questions. Prion amplification assays detect sublethal doses of prions that allow identification of nonclinical carriers of CWD (Wyckoff et al., 2015). Transgenic mice expressing cervid prion protein provide a source of prion-free animals with which to model indirect CWD transmission. Subtractive infectivity assays allow us to determine infectivity binding titers of soil and its components (Wyckoff et al., 2013). Highly efficient enrichment and fluorochrome labeling of prion amyloid allows monitoring prion movement and cellular and molecular interactions in live animals in real time. Here we show for the first time that soil naturally contaminated with prions can transmit CWD, estimate CWD prion infectious titers that soil can bind, and demonstrate that clay potentiates this binding capacity and dictates prion trafficking in live animals after ingestion.

## Materials and methods

### Mice

Animal work was approved by Colorado State University IACUC Protocol ID: 09-1580A, Approval Date: January 14, 2010 and the USDA/APHIS/WS-National Wildlife Research Center IACUC QA-1709. Tg(5037)cerPrP mice were generated as previously described (Angers et al., 2009).

### Prion isolates and strains

We used the CWD field isolates E2, sourced from a clinical Colorado captive elk, and D10, sourced from a clinical Colorado mule deer. We also used D10 serially passaged in Tg(5037)cerPrP mice as a biologically cloned prion strain, D10p2.

### Prion titer determination by bioassay and protein misfolding cyclic amplification (PMCA)

*Bioassay*. Tg(CerPrP)5037 mice express elk PrP^C^ and are susceptible to PrP^CWD^ infection by multiple routes (Angers et al., 2009; Michel et al., 2012a). Mice were used in this experiment as opposed to the natural host due to shorter incubation times, smaller space requirements, much lower cost, greater tolerance to handling (Groschup and Buschmann, 2008), feasibly larger group sizes and, most importantly, no *a priori* exposure to potential PrP^CWD^ environmental contamination. Additionally, we used Tg(CerPrP)5037 mice as a standardized susceptible host in which we can titer CWD prion strains and isolates to more accurately assess prion loads in animals and their excreta, estimate prion binding to soil and its components and compare prion titers among different strains and isolates. We confirmed the presence of cervid prions in all animals scored clinically ill with CWD by proteinase K (PK) digestion and western blotting (WB) or PMCA, if necessary, or immunohistochemistry (IHC) for PrP^Sc^ on a representative subset of animals, as described below.

We inoculated serial dilutions of E2 into Tg(CerPrP)5037 mice and plotted the corresponding days post inoculation (DPI) to terminal prion disease to generate survival curves as previously performed with other prion isolates (Prusiner et al., 1982; Brandner et al., 1996), (Figure S1A). We determined the dilution of E2 resulting in mortality of half the mice and expressed the infectivity as log_10_ lethal dose_50_ (LD_50_) units. We plotted DPI against infectivity titer (Figure S1B) and performed linear regression analysis to fit a curve that we used to estimate infectivity titers based on DPI as previously described by Reed and Munch (Reed, 1938).

*PMCA*. We performed PMCA as previously described (Pulford et al., 2012). Briefly, ten 30 μl replicates of each E2 dilution was mixed with 20 μl of NBH and incubated at 37°C for 30 min. We sonicated samples at 120 watts for 40 s in a Misonix 4000 sonicator horn and repeated the cycle for 24 h, constituting one PMCA round. For subsequent rounds we mixed 25 μl of each sample into 25 μl of fresh NBH. This process was repeated for six total rounds. Samples were digested with 50 μg/mL PK (Roche) for 30 min at 37°C, western blotted by electrophoresing them through a sodium dodceyl sulfate denaturing 10% polyacrilamide gel, electroblotting to PVDF membrane (Millipore) and probing with Bar-224 anti-prion protein antibody conjugated to horseradish peroxidase. We visualized PK-resistant PrP bands using chemiluminescent substrate and a Versadoc digital imaging system (Fuji).

We quantified E2 dilutions by PMCA by assigning them scores (in relative PMCA units, or RPUs) based on the PMCA round at which samples first become positive (Figure S1C, and as previously described (Pulford et al., 2012). PMCA successfully detected sublethal doses of E2 prions, demonstrating greatly increased sensitivity of this assay compared to bioassay. We then plotted PMCA scores vs. E2 prion titers to derive a second linear equation to estimate E2 prion titers in samples based on their PMCA scores (Figure S1D). We then compared infectivity titers determined by bioassay and PMCA scores to confirm reproducibility of prion titer estimation by both assays (Figure S1E). We used PMCA scores to predict the DPI to terminal illness of inoculated mice (Figure S1F) and inform us when we should expect onset of clinical signs of prion disease.

### Soil analyses

CWD-positive soils were collected with permission from Colorado Parks and Wildlife research pens in Fort Collins, CO and Wyoming Game and Fish wildlife research pens in Sybille, WY. These soils were from pens that housed CWD infected captive mule deer and elk, respectively. These soils will be referred to as “MD pen soil” and “elk pen soil.” Twelve 5-gallon buckets of soil were collected from the top 1-inch of pen soil. CWD negative soil used in this study was collected with permission from private property located in Southern Colorado game management unit 861 where CWD has not been detected in wild populations. All soil was autoclaved to eliminate ambient microbes to prevent illness and intercurrent death in mice.

Classification analysis of whole soil was conducted by the Colorado State University Soil, Water and Plant Testing Laboratory (Fort Collins, Co). X-ray diffraction (XRD) mineralogy analysis of whole soil was conducted by K-T GeoServices, Inc. (Gunnison, CO). Whole-soil analysis included XRD weight percentage for bulk (whole rock) and clay fraction (< 4 μm), pH, percent organic material, and soil texture classification of basic elements (Wyckoff et al., 2013). The following definitions were used for clay mineral classification: Mixed-Layer Illite/Smectite (M-L I/S)—A clay mineral group containing interlayered or interstratified Illite and Smectite. Illite and Mica—Common non-expanding minerals which are hydrated silicates containing potassium, silica and aluminum. Kaolinite and Chlorite—Common non-expanding hydrous aluminum silicate clay minerals.

### Soil ingestion estimates

Mice were placed on negative soil for 48 h then moved to a bedding free cage for another 48 h. Fecal matter was weighed to estimate average production per day, and food was weighed before and after 48 h to estimate average consumption. Samples of feces (before and after soil ingestion), soil and food were collected and analyzed by inductively coupled plasma resonance (ICP) by the Soil, Water and Plant Testing Laboratory, Colorado State University, Fort Collins, CO. Samples were analyzed for minerals including titanium, aluminum, and silica which have been used in previous soil ingestion studies. We used two similar methods for estimating soil ingestion based on mineral content of food, feces and soil samples as previously described (Mayland et al., 1975; Arthur and Alldredge, 1979; Beyer et al., 1994). The second method compared mineral content of feces from mice prior to being housed on soil to feces from mice after they were housed on soil for 48 h. We again looked at minerals that would have limited host uptake for the estimation.

### Chronic prion exposure

**Figure 1:**
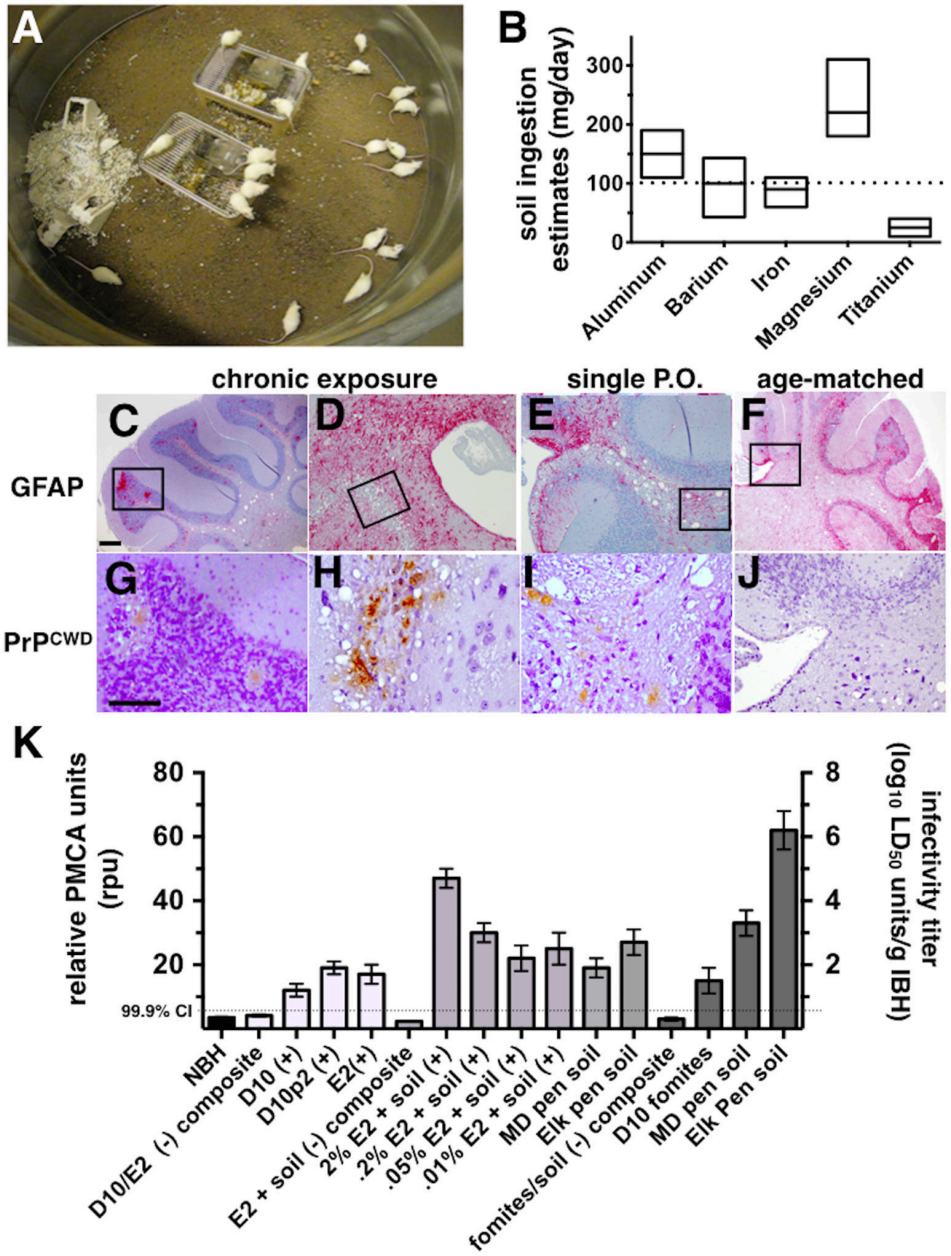
**CWD transmission to mice via soil-bound prions. (A)** Cohorts of mice were continuously housed for 1 year on soil naturally contaminated with CWD prions (*n* = 20 mice per soil type). **(B)** Soil ingestion by mice was estimated by analyzing feces for five different metals found in soil by two methods. Ranges for estimates calculated for each metal are shown. The dotted horizontal line indicates the median daily soil ingestion estimate. **(C–J)** Clinically sick mice exhibited characteristic prion neuropathology, including vacuolation, astrogliosis **(C–F)** and PrP^CWD^ deposition **(G–J)** in the cerebellum. Neuropathology appeared more severe in brains of mice chronically exposed to prions **(C,D,G,H)** than in mice orally infected with a single dose of prions pre-bound to soil **(E,I)** and age-matched controls **(F,J)**. Scale bars, 50 μm. **(K)** Significant prion titers were found in a subset of mice acutely treated (white bars, prions alone; light gray bars, prions bound to soil) or chronically exposed to prions naturally bound to soil or other contaminated fomites (dark gray bars). The dotted horizontal line indicates the 99.9% confidence interval for identifying positive samples by PMCA.

Mice were housed on one of the three soil-bedding treatment types (negative soil, MD pen soil and elk pen soil) in 4-foot round stock tanks with approximately 1 cm of soil bedding for 1 year (Figure 1A and Supplementary Video 1). Soil was exchanged every month for new pen soil. Used pen soil was decontaminated by alkaline digestion at the Colorado State University Diagnostic Laboratory. After 1 year mice were moved into traditional housing for an additional 235 days (600 dpi). We housed each of two cohorts of 20 mice on either MD or elk pen soil, and a cohort of 10 mice on negative control soil. All mice housed in stock tanks were female. Mice were given passive integrated transponder tags to keep individuals identified throughout the study. In a separate experiment, mice were housed on fomites (bedding, food and water) previously used by mice infected with E2 prions (*n* = 20 recipient mice) or uninfected mice (*n* = 10 recipients).

### Single p.o. inoculation

One hemisphere of cervid each brain was separately homogenized to a 10% dilution with sPMCA buffer (4 mM EDTA, 150 mM NaCl in PBS) in a commercial blender as previously described (Michel et al., 2012a) and aliquots were stored at −80°C until needed. E2 was the primary CWD isolate used in this study; D10 was used in previous studies.

Normal brain homogenate (NBH) was sourced from Tg(CerPrP)5037 mice and from a negative cow elk (eNBH) in Montana, a continuously negative population. Each were prepared as outlined above. We imported eNBH using the United States Veterinary Permit for Importation and Transportation of Controlled Materials and Organisms and Vectors, USDA # 110166 Research.

Negative soil for p.o. inoculation was microwaved for 5 min and UV irradiated for 1 h to kill environmental microbes. We serially diluted 10% E2 homogenate into 10% soil suspended in sucrose to resulting dilutions of 1:50 (2%), 1:500 (0.2%), 1:2000 (0.05%) and 1:10,000 (0.01%). We incubated mixtures at room temperature for 24 h to allow for prion adsorption to soil but avoid decomposition of brain homogenate. Seven different p.o. inoculation treatments were used including: 5 negative control mice p.o. inoculated with negative soil spiked with NBH, 4 cohorts of 45 mice inoculated with 1:50, 1:200, 1:2000, or 1:10,000 E2-soil, and two cohorts of 45 mice inoculated with either elk pen soil or MD pen soil. 12 h before p.o. inoculation water was withheld from mice to increase oral ingestion of inoculum. Mice received 50 μl p.o. of spike-soil-sucrose inoculum by pipette to allow for maximum exposure to oral lymphatic tissues. Mice were isolated for 5 min after p.o. inoculation to allow them to fully ingest inoculum before joining cage mates. P.o. treated mice were housed in standard housing with normal bedding for the duration of the study.

Negative soil inoculated mice (*n* = 8) were euthanized at 600 days post inoculation (dpi). We sampled mice from test cohorts using the strategy outlined in Table S1. Mice were euthanized independently of the schedule if they scored positive for clinical signs of terminal diseases including sudden weight loss, tail rigidity, ataxia, loss of extensor reflex, rough coat, kyphosis, akinesia and/or hyperactivity, or unrelated pain or distress that would be categorized as a USDA pain category of E.

### Detecting prion pathology and infectivity

Mouse brains were collected, sectioned and one hemisphere frozen at −80°C and the other fixed in 10% buffered formalin. We used sPMCA to detect PrP^CWD^ in frozen brains and feces after homogenization as previously described (7). Fixed tissues were assessed by histological techniques including H&E, GFAP, and IHC analysis (Michel et al., 2012a). Mice found positive by either sPMCA or IHC were considered CWD-positive, regardless of clinical signs.

### Biological or biochemical assay of subtracted infectivity from complex solutions (BASICS)

Subtracted infectivity assays were performed as previously described (Wyckoff et al., 2013). Briefly, indicated dilutions of NBH or E2 were mixed and incubated with indicated w/v proportions of soil and/or Mte at room temperature for 24 h. We centrifuged samples for 5 min @ 200 × g and inoculated 30 μl of each UV-irradiated supernatant supplemented with 1% Penicillin-Streptomycin intracranially into ten Tg5037(cerPrP) indicator mice per group. We also tested ten 30 μl aliquots of of each sample by PMCA. These scores were combined to determine mean prion binding affinities of soils and Mte.

### Live animal imaging

We monitored movement of fluorescent prions (un)bound to Mte in live animals as previously described (Michel et al., 2012b). Briefly, mice were inoculated p.o. with sucrose (*n* = 3) or fluorescent prions (*n* = 7) and visualized while anesthetized with 3 L/min Isofluorane in an IVIS 200 live animal imager (Caliper Life Sciences,Hopkinton, MA) at the indicated time points. We analyzed images using Living Image 4.0 software (Caliper).

### Flow cytometry

We prepared single cell suspensions of mouse tissue from stomach, small intestine, cecum, jejunum, Peyer's patches, spleen and mesenteric, mediastinal, axillary, inguinal, pharyngeal and popliteal lymph nodes by passing tissue samples through 45 μm nylon mesh screens in FACS buffer (0.1% BSA, 10 mM EDTA in 1XPBS). 10^6^ cells from each suspension were stained with antibodies and analyzed as previously described (Michel et al., 2012b). Briefly, we centrifuged cells at 250 × g for 5 min, washed twice in 1 mL FACS buffer, blocked Fc receptors using 2 ng/ml solution of Purified rat anti-mouse CD16/CD32 (BD Pharmingen) before staining with 50 μl of a 10 μg/mL solution of antibodies to distinguish neutrophils (Ly6G) macrophages (F4/80, Ly6C, CD11b), dendritic cells (CD11c), B cells (B220, CD21), T cells (CD3) and intestinal epithelial cells (CD24). Cells were washed twice following staining to remove unbound antibody and red blood cells were lysed by adding 1 ml of ACK buffer (150 mM NH_4_Cl, 10 mM KHCO_3_, 0.1 mM EDTA) to the cell pellet and immediately centrifuged at 250 × g for 5 min. The supernatant was removed and cell pellets were resuspended in 1X FACS buffer for analysis. Flow cytometry data was acquired using a DakoCytomation CyAnADP flow cytometer and analyzed using FlowJo version 8 (Tree Star, Ashland, OR). Nonparametric statistical analyses were performed using GraphPad Prism 5 (La Jolla, CA).

## Results

### Soil analysis

We analyzed five primary soils in this study, including those from CWD endemic states of Colorado and Illinois, prion-contaminated pens that housed mule deer in Colorado or elk in Wyoming, and soil from a non-CWD endemic state, Georgia. We classified Colorado (CO), mule deer (MD pen) and Georgia (GA) soils as sandy loam, elk pen soil as loamy silt, and Illinois (IL) soil as silty loam soil (Table S2). Clay content was comparable between soil types with overall clay content ranging from 11.5 to 19.8% of total soil weight. The smectite clays, which include Mte, were collectively measured and categorized as M-L I/S. Negative soils from CO and GA contained less smectite clay (19.1 and 19.7% of total clay, respectively) than MD pen (32.3%) and elk pen (27.4%) soils and IL soil from a CWD endemic area (43.3%). Soils primarily differed in quartz content, with the MD pen soil containing the highest quartz content at 66.6% weight compared to 35.9 and 29.3%. Soil pH varied from alkaline pH of the elk pen and IL soils at 8.9 and 8.0 pH, respectively, to the more neutral CO, GA and MD pen soils at 7.5. 7.2 and 7.0 pH, respectively. Additionally, the electrical conductivity, EC, a general measure of salinity in the sample, was 1.5 to 3.2 times higher in the elk pen soil than in all other soils.

### Transmission of CWD via soil-bound prions

Pens used to house mule deer and elk in CWD endemic areas of Colorado and Wyoming have been implicated in indirect transmission of CWD prions to these animals. However, the possibility of subclinical animals directly transmitting disease cannot be excluded. To determine whether soil from these pens contain prions that can transmit disease to susceptible animals, we continuously housed Tg5037(cerPrP) mice bred in prion free environments on pen soils for 1 year (Figure 1A). We estimated the amount of soil ingested per mouse per day to estimate potential daily oral prion doses mice might receive from soil. Soil ingestion estimates based on mineral measurements found in food, feces and soil showed variation, but contained a range of overlapping values with the exception of titanium (Figure 1B). Using these estimates we approximated a median daily soil ingestion estimate of 100 mg based on multiple minerals.

One of 20 mice housed on MD pen soil and two of 20 mice housed on elk pen soil exhibited clinical signs of terminal disease (Table 1), which was confirmed by observing CWD neuropathology, including vacuolation, astrogliosis and PrP^CWD^ deposition (Figures 1C,D,G,H) and PMCA. No mice progressed to terminal disease after acute exposure to a single oral dose of MD pen or elk pen soil (*n* = 90) or 2% infected brain homogenates (*n* = 39). When mixed with soil, 2 and 0.2% of the E2 CWD prion isolate, equivalent to approximately 10^9^ and 10^8^ LD_50_ units (Figures S1A,C), caused terminal CWD in 1/60 mice and 1/45 mice, respectively. These mice exhibited neuropathology (Figures 1E,I) that appeared more mild than that observed in chronically exposed mice, but more severe than age-matched controls (Figures 1F,J).

**Table 1:** **CWD infection detected by bioassay and PMCA**.

**Chronic Exposure**	**Clinical +/Total**	**sPMCA +/Total^b^**	**% Positive**
Negative soil	0/10	0/10	0
MD pen soil	1/20	3/20	15
Elk pen soil	2/20	4/20	20
Negative fomites^e^	0/10	0/10	0
D10 fomites	0/20	1/20	5
**SINGLE P.O. INOCULATION^a^**			
NBH	0/16	0/16	0
elk NBH	0/5	0/10	0
D10	0/17	1/17	5.9
D10p2	0/10	1/10	10
10^9^ E2	0/12	1/12	8.3
NBH + CO^c^ soil	0/8	0/8	0
10^9^ E2 + CO soil	2/60	13/60	21.7
10^8^ E2 + CO^c^ soil	1/45	6/45	13.3
5 × 10^7^ E2 + CO soil	0/45	5/45	11.1
10^7^ E2 + CO soil	0/45	2/45	4.4
MD pen soil^d^	0/45	3/45	6.7
Elk pen soil^d^	0/45	7/45	15.6

a *Unless otherwise indicated all inocula were a 2% dilution of brain homogenate*.

b *Includes clinical (+) samples confirmed by PMCA and/or PK digest and WB*.

c *CNE, Colorado soil from non-endemic area*.

d *The concentration of prions is unknown in pen soils*.

e *Bedding, food and water from previously used donor cages*.

We assayed all mice by PMCA to biochemically confirm CWD in clinically sick mice and assess subclinical infection in nonclinical mice. We identified two additional mice housed on MD pen soil and three mice housed on elk pen soil whose brains contained detectable amounts of prions (Table 1 and Figure 1K). We also identified 1/20 mice exposed to CWD prion-contaminated fomites, including food, water and cellulose-based bedding, with prions in its brain. We identified an additional 11 mice fed a single dose of 10^9^ LD_50_ units of E2 prions with CO soil, and five more mice fed 10^8^ LD_50_ units of prions, with prions in their brains. We detected prions in brains of 5/45 and 2/45 mice fed 5 × 10^7^ and 10^7^ LD_50_ units of E2 prions, respectively, with soil. Even without spiking, both MD pen and elk pen soils transmitted prions to 3/45 and 7/45 mice fed a single oral dose of each. Pre-binding 10^9^ LD_50_ E2 prions to CO soil significantly increased prion loads in orally infected mice (Figure 1K). Mice chronically exposed to unspiked MD pen and elk pen soils contained high prion titers in their brains, with elk pen-exposed mice harboring significantly higher prion loads than all other mice (6 ± 0.75 log_10_LD_50_, *n* ≥ 10, *p* < 0.01).

### Clay components in soil potentiated prion binding

**Figure 2:**
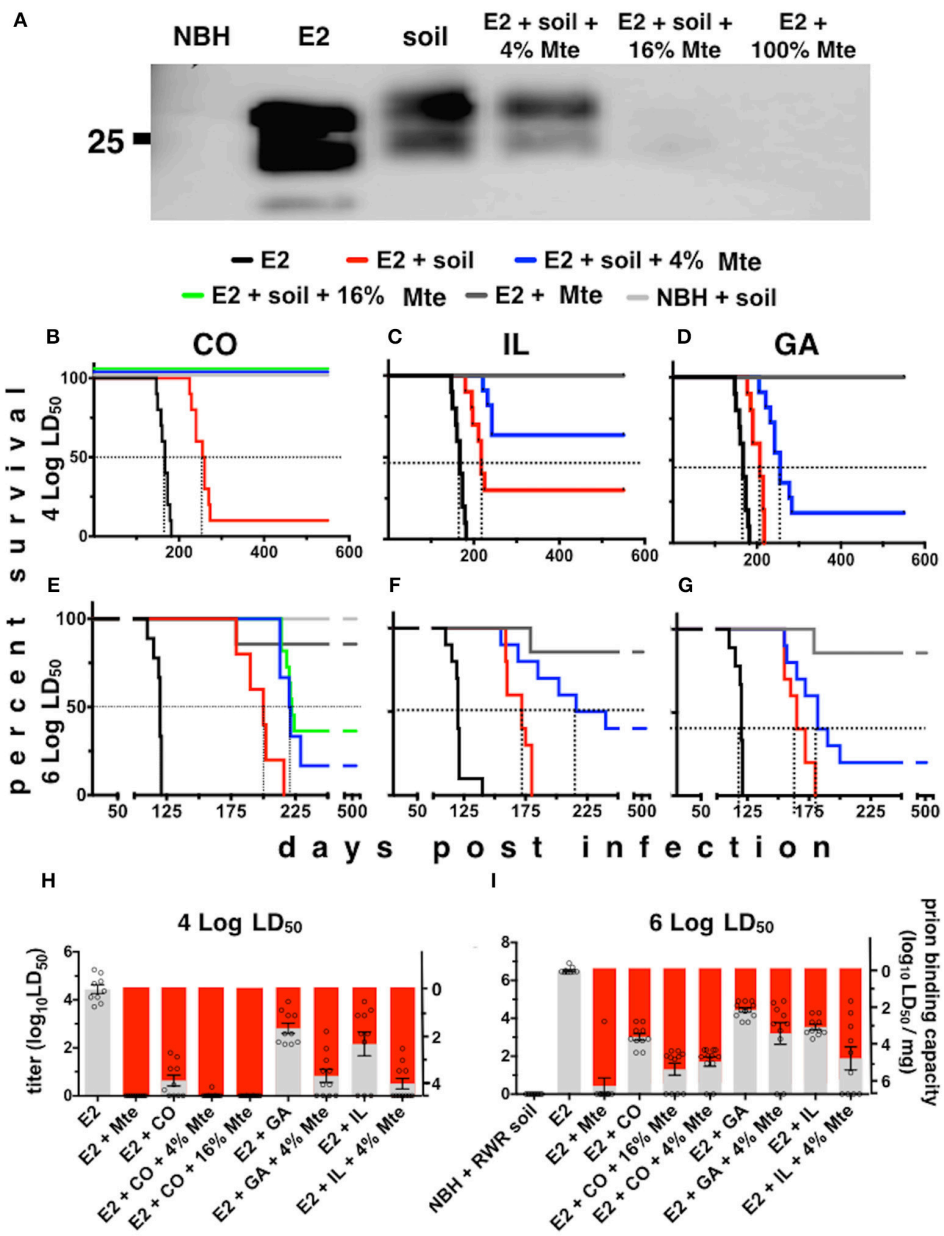
**Differential prion binding to different soils potentiated by clay particles. (A)** Western blot of supernatants from prions bound to various ratios of soil:Mte. Binding increases with increasing [Mte], which depletes prions from the supernatant. **(B–I)** Prion binding to soils from CWD endemic (CO, **B,E**; and IL, **C,F**) and non-endemic (GA, **D,G**) areas assessed by BASICS. Both low (10^4^ LD_50_ **B–D**) and high (10^6^ LD_50_ **E–G**) prion concentrations were assessed. All clinically sick animals were confirmed to harbor prions in their brains by PK digest and WB (data not shown). **(H,I)** Quantification of prion titers in supernatants (gray bars, left y-axes) and soil (red bars, right y-axes) by BASICS. Combined biological and biochemical BASICS scores reveal that at lower amounts of prions (**H**, 10^4^ LD_50_ units) CO soil bound nearly two logs more infectivity than IL soil and nearly three logs more than GA soil. CWD endemic soils (CO and IL) bound significantly more prions than non-endemic (GA) soil exposed to saturating amounts of prions (**I**, 10^6^ LD_50_ units, *p* < 0.05). Mte potentiates prion binding in the presence of all soils.

To estimate the prion binding capacity of soil and its clay components and the amount of prions adsorbed to soil in our acute and chronic exposure experiments, we expanded the use of our subtractive infectivity assay to CWD prions (Wyckoff et al., 2013). Calculating E2 infectivity titers enabled comparison of actual prion infectivity binding potential of soils containing different amounts of smectite clay. All soils tested bound E2 CWD prions (Figure 2A) similarly to mouse adapted scrapie prions that we previously reported (Wyckoff et al., 2013). Addition of increasing amounts of the smectite clay Mte increased prion binding, which depletes supernatants of detectable prions. Bioassays of supernatants from prion-soil matrices revealed significant differences in prion binding capacities of different soils. Mice inoculated with supernatants from prions pre-adsorbed to CO and IL soils lived significantly longer than mice inoculated with supernatants from prions pre-adsorbed to GA soil at both low (Figures 2B–D) and high (Figures 2E–G) titers of E2. Recall that soils from CWD endemic states CO and IL contained more clay than soil from GA, which is CWD-free. All clinically ill animals were confirmed to have CWD by detection of cervid prions in their brains by PK digest and Western blotting (data not shown). We also assayed supernatants for CWD prions by PMCA and combined these data with bioassay data to quantitatively compare prion binding capacity of each soil type. CO and IL soils bound over 10 times more infectious prions (6.3 ± 0.25 × 10^3^ and 6.26 ± 0.5 × 10^2^ LD_50_ units/mg soil, respectively) than GA soil (73 ± 15 LD_50_ units, *n* = 10, *p* < 0.05, Figures 2H,I). Addition of Mte potentiated binding of all soil types such that they all bound similar prion titers (*n* = 10). Because CO soil bound a mean of 6.3 × 10^3^ LD_50_ units of CWD prions/mg, we can now estimate that mice living on similar soil from MD and elk pens could have received up to 6.3 × 10^5^ LD_50_ units of prions per day, and 2.3 × 10^8^ prions for the year.

### Mte promotes prion uptake from the stomach and bioavailability to lymphoid organs

**Figure 3:**
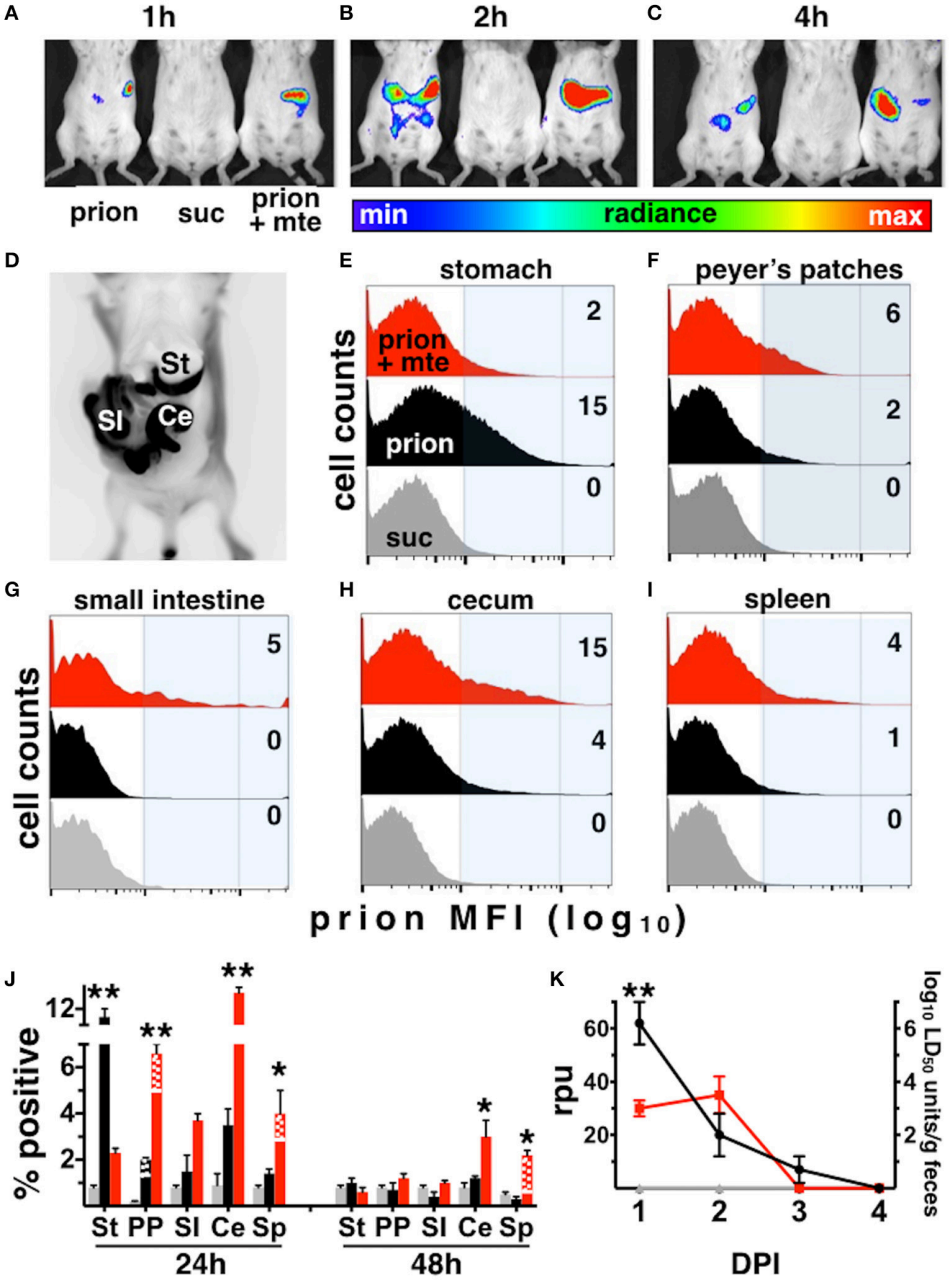
**Mte promotes retention and bioavailability of orally ingested prions. (A–D)** Mice retain fluorescent prions pre-bound to Mte longer than prions alone, mainly in areas consistent with the small intestine (SI) and cecum (Ce). **(E–I)** Flow cytometry reveals Mte promotes prion uptake from the stomach **(E)** into Peyer's patches **(F)** small intestine **(G)**, cecum **(H)** and spleen **(I)**. suc, sucrose only control **(J)** Significantly more cells in the stomach retain unbound prions (black bars) longer than if bound to Mte (red bars), which appears to facilitate prion transport from the stomach to macrophages (solid-colored bottom portion of bar) and B cells (checkered top portion) in Peyer's patches (PP) and spleen (Sp) and CD24+ epithelial cells from the small intestine (SI) and cecum (Ce). *n* = 5–8 mice per group ^**^ *p* < 0.01, ^*^ *p* < 0.05. **(K)** Mice fed unbound prions shed more prions in their feces (black line and circles) earlier and longer than mice fed Mte-bound prions (red line and circles).

The infection rates we observed for our Tg5037(cerPrP) mice appear similar to prevalence estimates of CWD in free-ranging cervid populations in the Rocky Mountain National Park and elsewhere (Wyckoff et al., 2015), validating these mice as a model for prion infection and transmission. We further used this model to investigate the influence of Mte on the kinetics and cellular mechanisms of CWD prion uptake, transport and shedding. Mice fed highly-enriched, fluorescent prions adsorbed to Mte retained more prions longer than mice fed unbound prions (Figures 3A–C). Mice retained Mte-prion complexes in organs and tissues consistent with the stomach and gastrointestinal tract (Figure 3D). Flow cytometry revealed significantly more unbound fluorescent prions (12 ± 1% positive cells, *n* = 8 mice) than bound (2.1 ± 0.2% cells, *n* = 5 mice) in the stomach after 24 h (*p* < 0.01, Figures 3E,J). More Mte-bound prions escaped the stomach to Peyer's patches (6.6 ± 1% prion^+^ cells (*n* = 8 mice) vs. 2 ± 0.1% cells with unbound prions (*n* = 5), Figures 3F,J), small intestine (not statistically significant, Figures 3G,J), cecum (12.5 ± 0.7% (*n* = 8) vs. 3.8 ± 1.2%, (*n* = 5), *p* ≤ 0.01, Figures 3H,J) and spleen (4.2 ± 0.2% (*n* = 8) vs. 1.3 ± 0.2%, (*n* = 5), *p* ≤ 0.05, Figures 3I,J) after 24 h. Significantly more Mte-prions persisted in the cecum (3 ± 0.9%, *n* = 8) and spleen (2 ± 0.1%, *n* = 8) at 48 h than unbound prions (1 ± 0.2% and 0.2 ± 0.1%, respectively, *p* < 0.05, *n* = 5, Figure 3J). Nearly all prions were associated with CD24-positive intestinal epithelial cells, which includes M cells. Predominantly CD11b/F4/80+ macrophages took up prions in lymph nodes and spleen at 24 h (70 and 75% of total prion^+^ cells, respectively), but mostly CD21/35+ B cells harbored prions in the spleen by 48 h (88% of total). PMCA of mouse fecal prions showed that mice retained approximately 300 times more Mte-bound prions than unbound prions, more of which mice shed earlier and longer (3 ± 1 × 10^6^ LD_50_ units of bound prions (*n* = 5) vs. 1 ± 0.3 × 10^9^ LD_50_ units unbound prions (*n* = 8), *p* < 0.01, Figure 3K).

## Discussion

Here we report that chronic exposure to naturally contaminated soil is sufficient for CWD transmission in prion susceptible mice. This was previously shown in captive mule deer, a natural host species. However, direct transmission from nonclinical animals in endemic areas could not be definitively excluded and indirect transmission from environmental sources remained a poorly quantified component of CWD transmission. We have demonstrated, for the first time, that soil from pens that previously housed prion infected animals transmitted CWD prions to susceptible transgenic mice bred in prion-free conditions. We observed terminal clinical signs and classic CWD neuropathology in 3/40 mice continuously housed for 1 year on prion-contaminated MD or elk pen soil. We also detected subclinical prion infection in an additional five mice, resulting in transmission rates comparable to prevalence estimates for natural cervid hosts in CWD endemic areas. These data confirm that these pen soils contain infectious prions that can be indirectly transmitted, and that Tg(cerPrP)5037 mice can accurately model indirect CWD transmission.

This study also supports the debated hypothesis that CWD prions bound to soil are more infectious (Johnson et al., 2007) than prions without soil. None of the 39 mice orally inoculated with three unique CWD isolates demonstrated clinical illness. However, 3/105 mice inoculated with the equal or lower doses of CWD prions plus soil progressed to terminal clinical disease. PMCA revealed significantly more mice with subclinical levels of CWD prions when fed prions with soil than with prions alone. We also observed a higher percentage of subclinical mice after feeding them a single dose of MD pen and elk pen soil (11.1%, *n* = 90) than mice fed high doses of unbound CWD prion isolates (7.7%, *n* = 39) and comparable to percentages of subclinical mice fed 10^8^ and 5 × 10^7^ LD_50_ units of E2 with soil (12.2%, *n* = 90). These observations also provide an estimate of the prion titers contained in those pen soils.

Moreover, we detected significantly higher prion titers in brains of subclinical mice acutely and chronically exposed to prions with soil than mice fed unbound CWD prions. Overall, the proportion of mice testing positive for CWD by PMCA after a single oral dose of prions with soil (4.4–21.7%) were comparable to infection rates of mice continuously housed on the pen soils (15–20%). These proportions are far lower than has been reported for natural hosts housed in the pens from which we sourced the soil (Miller and Williams, 2003). However, cervids in those pens inhabited those pens their entire lives, in contrast to mice housed on soil for just 1 year. No data exists for the prevalence of infected cervids after the first year in those pens. Different digestive physiology in mice, which have simple stomachs, vs. the four-chambered stomachs of cervids and sheep, could also contribute to differences in incidence.

Typical experimental challenge studies use a single dose of infectious material, but we agree with Williams et al. (Miller and Williams, 2004; Williams, 2005) that this is not representative of a natural course of infection and likely underestimates the timeframe of a typical natural infection. Instead the total lifetime exposure dose directly influences the disease time course and possibly intensity. Thus, mice housed on contaminated soil were more likely to become infected, and to contract clinical disease, than mice treated with the same soil in a single p.o. dose.

The difference observed between the chronic exposure treatments of MD pen soil and the elk soil could be due to titer differences. The Wyoming elk pens routinely have new cases of CWD in captive elk, while the Colorado mule deer pens have been housing fewer deer and noticed a decline in new cases within the captive deer. Alternatively, prevalence differences could be due to CWD prion strain differences or host PrP sequence. The mice used in this study were transgenic for the elk *Prnp* gene, and incidence was highest in mice housed on the elk pen soil as compared to the MD pen soil. The difference between incidences at facilities could be dependent on soil differences. All soils tested here exhibited increasing prion binding capacity with increasing clay content that we hypothesize dictates environmental stability. Our experiments showing Mte potentiated soil binding to prions, as well as evidence demonstrating increased infectivity of soil bound prions (Johnson et al., 2007) and correlations of soil clay content with disease prevalence (Walter et al., 2011) supports this hypothesis. We have successfully used our transgenic mouse chronic exposure model and BASICS to measure environmental contamination, degradation rates, and rates of transmission over time. We can further apply these tools to forecast and/or estimate the ecology of the disease at the population and landscape level.

Even less is known about how soil influences prion uptake, retention, replication and infection in affected animals. Here we report that mice absorbed prions bound to soil significantly faster from the stomach and retained soil-bound prions significantly longer in the cecum, lymph nodes and spleen than unbound prions. Soil likely facilitated prion transport from the stomach to the small intestine, where we showed CD24 positive epithelial cells, including enterocytes and M cells previously shown to take up prions (Foster and Macpherson, 2010; Da Costa Dias et al., 2011; Donaldson et al., 2012; Kincaid et al., 2012), retain prions longer than unbound prions. Later, soil-bound prions were captured mainly by CD11b^+^/F4/80^+^ macrophages in Peyer's patches and spleen. Macrophages likely transfer soil-bound prions to CD21/35^+^ B cells, which can transport them to germinal centers for robust replication by follicular dendritic cells, as we have shown previously (Michel et al., 2012b). Increased detection of unbound prions excreted into feces compared to soil bound prions confirmed retention in the gut, lymph nodes and spleen. Increased retention likely potentiates early replication, later dissemination and ultimately neuroinvasion, which leads to increased neuronal replication and progression to terminal CWD. Thus, soil can increase both environmental stability and bioavailability of prions that it binds.

These findings have implications for human exposure to, and facilitated uptake of CWD prions on the landscape. Soil likely facilitates inhalation and even ingestion of prions by hunters, hikers and wildlife professionals in the environment. A significant species barrier exists that limits prion disease transmission from cervids to humans (Miller and Williams, 2004; Williams, 2005). But soil may alter this barrier and facilitate rapid and efficient absorption of prions into mucosal tissues that drain into lymphoid tissue where zoonotic prion replication could occur. We are currently exploring this possibility.

Long-term environmental reservoirs of pathogens contribute to the epidemiology of many diseases including avian influenza, anthrax, hanta virus and botulism (Kallio et al., 2006; Rohani et al., 2009). Quantifying titers and persistence of PrP^CWD^ in the environment has been particularly challenging due to limited sensitivity of existing laboratory assays. Our findings significantly advance our understanding of the influence of soil on environmental prion exposure and transmission, and the pathobiology of prions ingested by animals in this context.

## Author contributions

MZ is the principal investigator who designed, performed and interpreted experiments, and wrote and edited the paper. AW conceived the project and designed, performed and interpreted experiments, and wrote and edited the paper. SK performed and interpreted experiments and edited the paper. KL and JS performed experiments. BM, DH, AO, and MRM performed and interpreted experiments, and edited the paper. GT and MWM designed experiments, provided reagents and wrote and edited the paper. KV conceived the project, designed and interpreted experiments and wrote and edited the paper.

### Conflict of interest statement

The authors declare that the research was conducted in the absence of any commercial or financial relationships that could be construed as a potential conflict of interest.
