# Supplementary material for: Clay Components in Soil Dictate Environmental Stability and Bioavailability of Cervid Prions in Mice
Source: Front Microbiol. 2016 Nov 23;7:1885. doi: 10.3389/fmicb.2016.01885 (PMC5120086; doi:10.3389/fmicb.2016.01885)
Supplement: Supplementary file 2 [file DataSheet1.DOC]

**Clay components in soil dictate environmental stability and bioavailability of chronic wasting disease prions**

A. Christy Wyckoff^1^, Sarah Kane^1^, Krista Lockwood^1^, Jeff Seigman^1^, Brady Michel^1^, Dana Hill^1^, Aimee Ortega^1^, Mihnea R. Mangalea ^1^, Glenn Telling^1^,Michael W. Miller^2^, Kurt Vercauteren^3^ and Mark D. Zabel^1^

^1^Prion Research Center at Colorado State University, Department of Microbiology, Immunology and Pathology, College of Veterinary Medicine and Biomedical Sciences, Fort Collins, CO 80523, USA

^2^Colorado Parks and Wildlife, 317 W. Prospect Road, Fort Collins, CO 80523, USA

^3^National Wildlife Research Center (NWRC), Wildlife Services, United States Department of Agriculture, La Porte Avenue, Fort Collins, CO 80521, USA

**Supplementary Information**

This supplementary information includes

Figure S1

Table S1

Table S2

Prion Soil movie


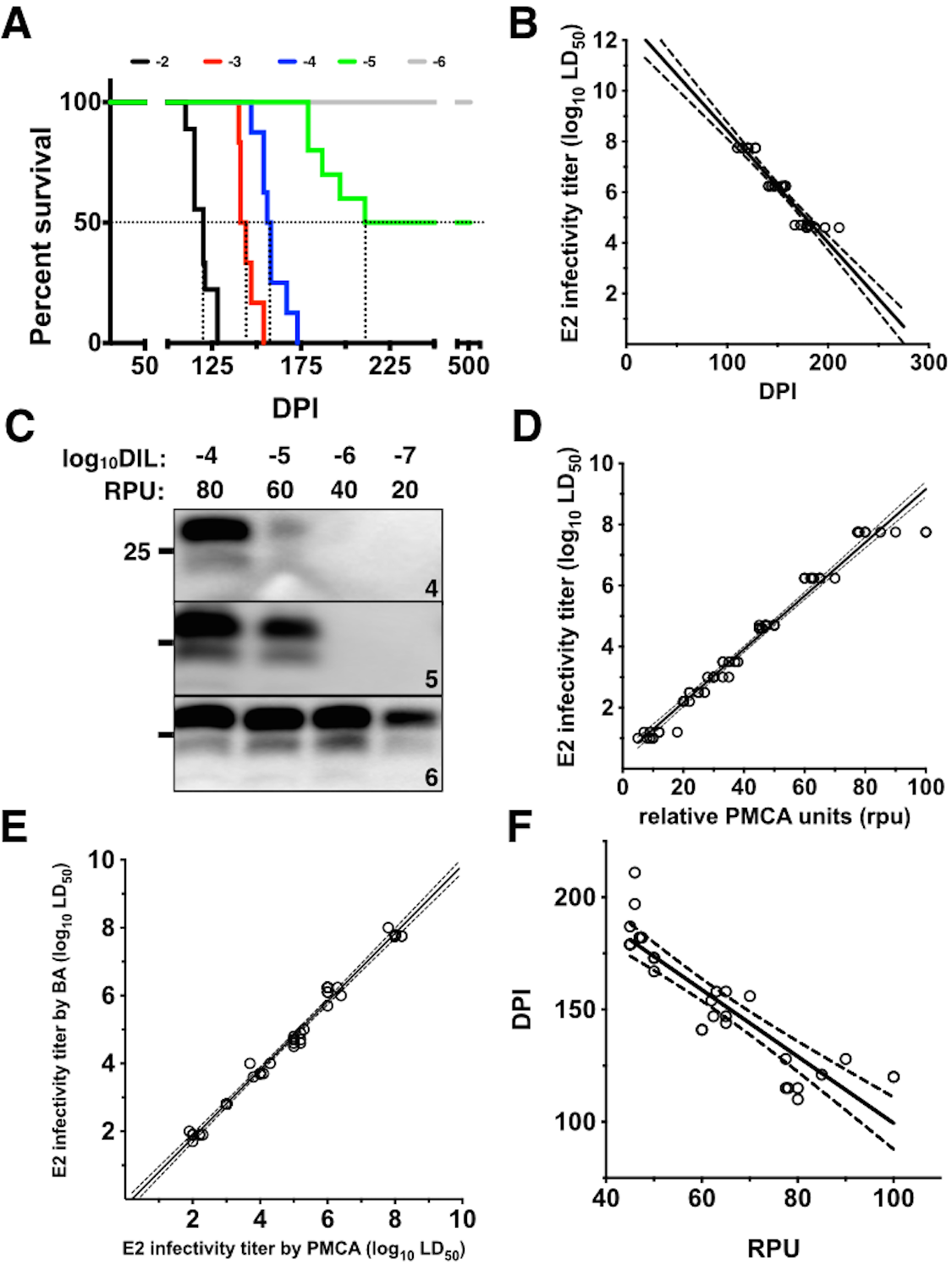


**Figure S1. E2 prion titer determination. (**A) Bioassay of E2 in susceptible Tg5037(cerPrP) mice. Cohorts of ten mice were inoculated i.c. with the indicated log_10_ dilution of E2 brain homogenate and survival days post infection (DPI) monitored. B) Rounds 4, 5 and 6 of PMCA show amplification of E2 at the indicated dilution and the resulting PMCA score in relative PMCA units (RPU). Markers to the left of each blot indicate the molecular weight in kilodaltons. (C) Infectivity titers for E2 were determined by bioassay using the Reed-Muensch method. Linear regression was used to correlate infectivity titer to DPI (y = 12.85 - 0.04422*x*, R^2^ = 0.9117, Sy.x = 0.3954) and (D) RPU (y = 0.4207 - 0.0.08735*x*, R^2^ = 0.9702, Sy.x = 0.3856). (E) Titers calculated by both methods were compared to assess reproducibility of PMCA (R^2^ = 0.9884). Infectivity titers for E2 dilutions that did not cause terminal disease were extrapolated from the equation in (C) for bioassay. (F) Correlation of RPU to DPI was assessed and used to predict onset of clinical disease in infected mice (y = 247.9 - 1.485*x*, R^2^ = 0.7990, Sy.x = 12.89).

| Table S1. Single P.O. Treatments | | | | | | | | | | | | | |  | |
| --- | --- | --- | --- | --- | --- | --- | --- | --- | --- | --- | --- | --- | --- | --- | --- |
| dpi | Negatives^a^ | | | E2^b^ | D10^c^ | Soil spiked with E2 CWD dilutions | | | | | | Naturally contaminated soil | |  | |
|  | NBH | eNBH | NBH soil^d^ | 1:50 | 1:50 | 1:50^e^ | 1:50^f^ | 1:200 | 1:2,000 | 1:10,000 | Elk pen | | MD pen | |  |
| 0 d | 16 | 5 | 8 | 12 | 17 | 15 | 45 | 45 | 45 | 45 | 45 | | 45 | |  |
| 50 d | 16 | 5 | 8 | 12 | 17 | 15 | 35 | 35 | 35 | 35 | 35 | | 35 | |  |
| 200 d | 16 | 5 | 8 | 12 | 17 | 15 | 20 | 20 | 20 | 20 | 20 | | 20 | |  |
| 600 d | 0 | | | | | | | | | | | | |  | |
| ^a^ Negative brain homogenates  ^b^ CWD elk isolate E2  ^c^ CWD mule deer isolate D10  ^d^ NBH spiked into negative soil from Southern Colorado  ^e^ Pilot study using E2 spiked into soil from the Front Range  ^f^ E2 spiked into negative soil from Southern Colorado | | | | | | | | | | | | | | | |

| Table S2. Soil analysis | | | | | |
| --- | --- | --- | --- | --- | --- |
| Mineral | CO | MD Pen | Elk Pen | GA | IL |
| Quartz | 35.9^a^ | 66.6 | 26.3 | 28.8 | 23.4 |
| K-Feldspar | 9.3 | 3.2 | 4.0 | 5.5 | 5.3 |
| Plagioclase | 38.3 | 5.7 | 49.5 | 35.5 | 37.1 |
| Amphibole | 1.3 | 1.0 | 1.6 | 1.3 | 1.7 |
| Calcite | 1.3 | 1.1 | 2.8 | 1.5 | 2.2 |
| Pyrite | 1.6 | 2.6 | 1.2 | 0.9 | 0.0 |
| Hematite | 0.8 | 0.0 | 0.0 | 0.0 | 0.0 |
| R0 M-L I/S 90S^b^ | 2.2 (19.1)^c^ | 6.4 (32.3) | 4.0 (27.4) | 1.5(19.7) | 4.2(43.3) |
| Illite & Mica^b^ | 7.7 (67.0) | 11.2 (56.6) | 8.5 (58.2) | 4.3(55.6) | 5(51.5) |
| Kaolinite^b^ | 1.4 (12.2) | 1.6 (8.1) | 1.4 (9.6) | 1.7(22.1) | 0.3(3.1) |
| Chlorite^b^ | 0.2 (1.7) | 0.6 (3.0) | 0.7 (4.8) | 0.2(2.6) | 0.2(2.1) |
| Total | 11.5 (100) | 19.8(100) | 14.6(100) | 7.7(100) | 9.7(100) |
| Soil Characteristics | | | |  |  |
| Texture class | Sandy Loam | Sandy Loam | Loamy Sand | Sandy Loam | Silty Loam |
| % Sand | 72.0 | 74.0 | 80.0 | 74.0 | 35 |
| % Silt | 14.0 | 10.0 | 8.0 | 16.0 | 55 |
| % Clay | 14.0 | 16.0 | 12.0 | 8.0 | 10 |
| Ph | 7.5 | 7.0 | 8.9 | 7.2 | 8.0 |
| EC (mmhos/cm)^d^ | 4.6 | 3.5 | 11.6 | 7.5 | 6.7 |
| % Organic Material | 3.6 | 4.6 | 9.9 | 11.1 | 24.4 |
| ^a^ % weight of whole soil  ^b^ clay classification  ^c^ % of total soil (% total clay weight)  ^d^ electrical conductivity (EC), measurement of salinity | | | |  |  |
